# Design and Implementation of a YOLOv2 Accelerator on a Zynq-7000 FPGA

**DOI:** 10.3390/s25206359

**Published:** 2025-10-14

**Authors:** Huimin Kim, Tae-Kyoung Kim

**Affiliations:** Department of Electronic Engineering, Gachon University, Seongnam 13120, Republic of Korea; kyle225@gachon.ac.kr

**Keywords:** You Only Look Once, hardware accelerator, object detection, convolutional neural network, field-programmable gate array

## Abstract

You Only Look Once (YOLO) is a convolutional neural network-based object detection algorithm widely used in real-time vision applications. However, its high computational demand leads to significant power consumption and cost when deployed in graphics processing units. Field-programmable gate arrays offer a low-power alternative. However, their efficient implementation requires architecture-level optimization tailored to limited device resources. This study presents an optimized YOLOv2 accelerator for the Zynq-7000 system-on-chip (SoC). The design employs 16-bit integer quantization, a filter reuse structure, an input feature map reuse scheme using a line buffer, and tiling parameter optimization for the convolution and max pooling layers to maximize resource efficiency. In addition, a stall-based control mechanism is introduced to prevent structural hazards in the pipeline. The proposed accelerator was implemented on the Zynq-7000 SoC board, and a system-level evaluation confirmed a negligible accuracy drop of only 0.2% compared with the 32-bit floating-point baseline. Compared with previous YOLO accelerators on the same SoC, the design achieved up to 26% and 15% reductions in flip-flop and digital signal processor usage, respectively. This result demonstrates feasible deployment on XC7Z020 with DSP 57.27% and FF 16.55% utilization.

## 1. Introduction

Object detection is a deep learning model designed to identify and localize objects in images, typically employing a convolutional neural network (CNN) architecture trained on large-scale datasets. Since the emergence of such datasets and high-performance computing hardware [1,2,3], extensive research has been conducted to advance object detection [4,5]. Early studies primarily focused on improving accuracy by applying CNN operations to proposed regions of an image for feature extraction [6]. Subsequent studies enhanced the inference speed through one-stage methods that perform a single CNN operation across the entire image [7,8,9].

Among various one-stage approaches, the You Only Look Once (YOLO) framework has emerged as one of the most prominent and extensively studied. Over successive generations, the YOLO family has incorporated architectural enhancements such as feature pyramid and path aggregation networks [10,11,12]. More recent variants illustrate diverse design directions: YOLO-NAS prioritizes inference efficiency and quantization optimization, while YOLOv8 emphasizes top-tier accuracy and scalability through its range of model sizes [12]. Additionally, YOLO-World integrates both image and text inputs into a multimodal detection framework [9]. These advancements exemplify the continued evolution of the YOLO family [9,12] and have heightened the demand for efficient hardware acceleration across diverse deployment scenarios. Given the limitations of graphics processing units in terms of power consumption and cost, particularly for embedded and edge applications, field-programmable gate array (FPGA)-based YOLO accelerators have emerged as a promising alternative.

Research on FPGA-based YOLO accelerators has applied several optimization techniques to maximize the efficiency of parallel processing and pipelining. Quantization is among the most widely adopted methods because it reduces the memory footprint and bandwidth requirements [13,14,15,16]. Previous studies explored various bit precisions tailored to application goals, including 4-bit quantization to minimize latency [17,18,19] and 16-bit quantization to maintain accuracy [20,21,22]. More recently, a design applying 8-bit quantization to data and 5-bit quantization to weights enabled two multiplication operations within a single digital signal processor (DSP) slice [23].

Local memory data reuse is another critical strategy for reducing off-chip memory access and improving energy efficiency [24]. Examples include filter data reuse in block random access memory (BRAM) [25], output data reuse in registers [26], and input data reuse using line buffers [27,28]. Tiling techniques partition the filter and input feature map (IFM) data into smaller tiles for partial on-chip processing. These techniques have been combined with multi-filter parallelism to improve the performance of YOLOv6 [29] and YOLOv2 [30]. Tiling has also enhanced resource utilization in general matrix multiplication (GEMM)-based architectures [31]. Additional algorithmic optimizations include employing the Winograd algorithm to simplify convolutional operations and reduce resource usage in YOLOv2 accelerators [32], and implementing classification acceleration structures using parallel support vector regression [33].

### 1.1. Motivations

Despite recent advancements, most prior studies have primarily targeted high-performance system-on-chip (SoC) platforms to maximize throughput. While such architectures are valuable, there is a growing need for lightweight accelerator designs that can operate efficiently on resource-limited SoCs. Compact devices, in particular, impose strict constraints on size and power consumption, making small-scale and resource-efficient systems highly desirable. To address this challenge, the present study proposes a resource-efficient accelerator optimized for the Xilinx Zynq-7000 (XC7Z020), a representative low-spec SoC. In addition, we focus on YOLOv2 as the target model since its fundamental operations such as 3×3 and 1×1 convolutions with stride = 1 and max pooling remain the core computational blocks in subsequent YOLO versions.

We propose a lightweight accelerator architecture that incorporates optimized quantization, data reuse, tiling parameters, and a pipeline controller to enable efficient computation in such an environment. The operational process of the proposed architecture is described using a finite state machine (FSM), and its efficiency is validated through system-level evaluation.

The primary contributions of this study are as follows:

- We optimize the filter strategy and tiling parameters to address the constraints of resource-limited environments. We optimize the data reuse and pipeline architecture for resource-constrained environments. In particular, we adopt 16-bit integer (INT16) quantization to efficiently utilize the limited BRAM on the target platform. This enables the implementation of an optimized filter reuse structure using 25.6 KB of BRAM and a line-buffer-based IFM reuse structure using 153.6 KB of BRAM. In addition, we define tiling parameters that minimize the hardware complexity and reconfigure the pipeline controller using a stall mechanism to ensure continuous data flow.
- We propose a complete accelerator architecture built on the optimized structures. The optimization particularly involves hardware parameters that determine the BRAM size. The architecture comprises six controllers and nine processing units, with its operational flow systematically described through the FSM states of the main controller—Idle, Start, MP, Conv, and Done—demonstrating efficient control of the convolution and max pooling operations.
- We implement the proposed accelerator on an XC7Z020 SoC and perform system-level evaluation to verify its efficiency. The experimental results show that INT16 quantization yields a negligible accuracy loss of approximately 0.2% compared with the 32-bit floating-point (FP32) baseline. Furthermore, compared with other accelerators implemented on the same SoC, our design achieves superior resource efficiency, reducing flip-flop (FF) and DSP usage by up to 26% and 15%, respectively.

### 1.2. Organization

The remainder of this paper is organized as follows. Section 2 describes the microarchitecture and advanced extensible interface (AXI) interconnect used in the design. Section 3 details the proposed optimization methods and structures, as well as the complete accelerator architecture. Section 4 presents the implementation results, system-level validation, and comparative analysis with previous studies. Finally, Section 5 concludes the paper by summarizing our contributions and results.

## 2. Hardware Architecture

This section details the microarchitecture of the considered design and outlines the direct memory access (DMA) operation over the AXI in Zynq SoCs, which incorporate an embedded processing system (PS).

### 2.1. Microarchitecture

**Figure 1:**
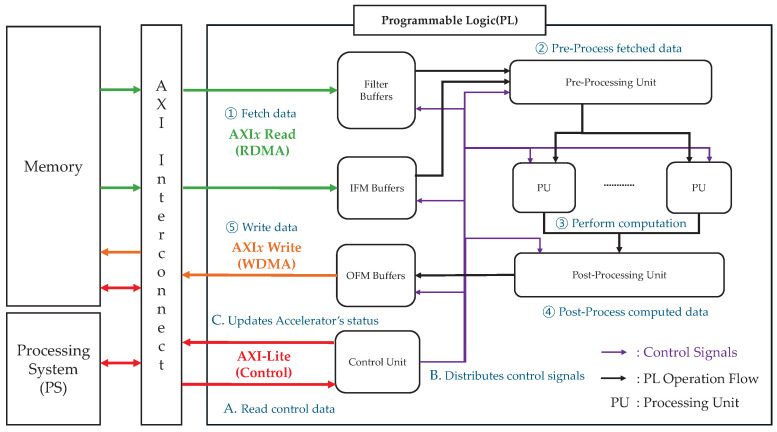
Block diagram of a sliding window-based microarchitecture using AXI interconnect.

This study employs a sliding window-based accelerator instead of adopting a resource-intensive GEMM-based approach. Figure 1 depicts the sliding window-based microarchitecture implemented on an FPGA [30]. The architecture comprises the programmable logic (PL), PS, and external memory. The PL contains a filter, IFM, and output feature map (OFM) buffers, along with a pre-processing unit, processing units (PUs), a post-processing unit, and a control unit.

The operational process within the PL for writing OFM data to the memory proceeds as follows:

- ① The filter and IFM data stored in the external memory are fetched via read direct memory access (RDMA) over the AXI interconnect and stored in on-chip buffers for reuse and tiling.
- ② The pre-processing unit processes the fetched data and forwards it to the PUs.
- ③ Each PU handles a specific data segment and begins its execution as soon as its data becomes available. Computations within each PU are performed in a parallel and pipelined manner. When applicable, the results of filter operations are accumulated in the internal accumulator of the PU.
- ④ After completing its operations, each PU transfers its output to the post-processing unit, which further processes the data and stores it in the OFM buffer.
- ⑤ Once sufficient output data has been accumulated in the OFM buffer, it is written back to the external memory via write direct memory access (WDMA) over the AXI interconnect.

The control unit exchanges control signals with the PS, as discussed in detail in Section 2.2.

### 2.2. Zynq-7000 AXI Interconnect

The Zynq-7000 SoC uses an AXI interconnect to facilitate communication between the PS, PL, and external memory. The AXI standard provides several interface types, including the high-performance AXI*x* (where *x* = 3 or 4) for continuous high-speed data transfers, AXI-Lite for low-resource control transactions, and AXI-Stream for continuous data processing without address information [34].

Within the PL, the parallel and pipelined architectures require large volumes of data—such as IFMs, OFMs, and filter weights—to be transferred continuously at a high speed from specific memory addresses. This is achieved via DMA transfers between the IFM, OFM, and filter buffers in the PL and the main memory through a high-performance AXI*x* interface. By contrast, the control data from the PS to the PL control unit are read only once per layer, and the control unit periodically sends simple monitoring data back to the PS. Consequently, AXI-Lite is used for these control transactions.

Each buffer reads and writes data through its assigned AXI channels, as shown in Figure 1. From an AXI perspective, the operational process is as follows:

- **A** Via AXI-Lite, the PS sends control data—such as current layer information, target memory addresses for each buffer’s DMA operation, and accelerator status—to the control unit.
- **B** The control unit distributes appropriate control signals to each buffer and PU based on this information, initiating the accelerator’s operation.
- **C** Subsequently, the control unit continuously updates the accelerator’s status and reports it back to the PS, enabling real-time monitoring.

## 3. Proposed Architecture

This section presents the YOLOv2 accelerator, which adopts a sliding window-based microarchitecture and leverages the AXI interconnect. We begin by outlining the resource constraints of the target SoC, the XC7Z020, because these limitations directly shape the architectural decisions. Based on these constraints, we describe the quantization and data reuse techniques employed to reduce the PL–memory bandwidth, followed by the configuration of the tiling parameters and corresponding processing flow designed to maximize hardware utilization. Finally, we introduce a stall-based pipeline control method integrated with the tiling structure.

Table 1 summarizes the symbols used throughout this study for clarity.

### 3.1. Constraints

Optimizing the quantization method, data reuse strategy, and tiling parameters based on the resource constraints of the device is necessary to efficiently implement the YOLOv2 accelerator on the target SoC, the XC7Z020. The XC7Z020 provides 53,200 look-up tables (LUTs), 106,400 FFs, 630 KB of BRAM, and 220 DSP slices. The hardware resource definitions used in this work are summarized in Table 2.

In this study, we adopt 16-bit integer (INT16) quantization to maintain an accuracy comparable to floating-point while improving performance. Table 3 lists the calculated maximum data sizes for the IFM, bias, and weights in each YOLOv2 layer for 16-bit and 32-bit representations. In the FP32 case, the large data size increases the memory–PL bandwidth requirements, whereas the complexity of floating-point arithmetic imposes greater demands on the computational resources. Given the characteristics of the low-resource XC7Z020 board, which operates at clock frequencies in the MHz range, INT16 quantization is essential to reduce bandwidth usage and simplify computation.

Beyond INT16 quantization, an efficient data reuse method with low resource overhead is necessary to further reduce the energy consumption and bandwidth between the memory and PL. For YOLOv2, the maximum data size—excluding bias—can reach the megabyte scale (see Table 3). Consequently, storing all data in FFs or BRAM for reuse is infeasible on the XC7Z020. The proposed design addresses this issue by reusing the filter and IFM data through BRAM-based buffering, as detailed in Section 3.2 and Section 3.3.

XC7Z020 also requires optimized tiling parameters to minimize the LUT, FF, and DSP usage. In YOLOv2, the IFM size ($I_{s}$) varies by layer. In addition, implementing a control scheme that manages unused tiles for each layer would waste significant FF and LUT resources. For example, during convolution, as the tiling parameters $T_{r}$ and $T_{c}$ increase, the number of FFs required for the tile storage ($FF_{\mathrm{tile}}$) also increases, as shown in (1). Therefore, the tiling parameters must be configured to maintain low hardware complexity for both convolution and max pooling operations. These configurations are discussed in Section 3.4 and Section 3.5.

(1) $$FF_{\mathrm{tile}}=T_{r}\times T_{c}\times Q_{b}\times 8.$$

The tiling parameters ($T_{r}$, $T_{c}$) must be determined by considering not only the $FF_{\mathrm{tile}}$ but also the DSP resources. The DSP slices in the XC7Z020 are equipped with a 25 × 18-bit multiplier and an accumulator. The 16-bit quantized operations used in this architecture can be processed by mapping one MAC (Multiply–Accumulate) operation to a single DSP slice. Therefore, $T_{r}$ and $T_{c}$ must be configured to maintain low overall hardware complexity for both convolution and max pooling operations, taking into account the given constraints on LUT, FF, and DSP resources. These specific parameter configurations are discussed in Section 3.4 and Section 3.5.

### 3.2. Filter Reuse

We adopt a BRAM-based filter reuse strategy for efficient data handling. As shown in Table 3, the total maximum weight data for a 16-bit representation amounts to 23.59 MB, whereas the size of a single filter set is only 23.04 KB. Because the combined size of all the bias data (2.56 KB) and a single set of filter weights (23.04 KB) fits within the available BRAM resources of the XC7Z020, filter reuse can be implemented entirely on-chip.

**Figure 2:**
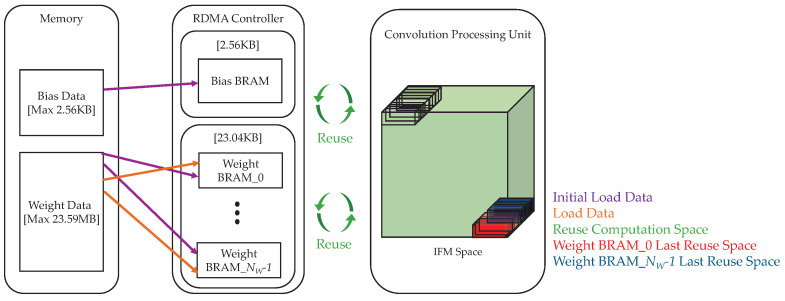
Process of loading bias and weight data and reusing them within a filter reuse-based accelerator architecture.

As shown in Figure 2, the BRAM controller first loads all the bias data for the current layer (up to 2.56 KB), followed by the weight data. The bias data remain unchanged until the next layer begins, whereas the weight data—either the entire set or a portion of it (up to 23.04 KB)—are loaded depending on the layer. This 23.04 KB space is divided among multiple weight BRAMs. Importantly, weight pre-processing can begin before all weights are fully loaded, allowing computation to begin as soon as the initial data are available.

The IFM space denotes the region accessed by the PU during computation. In Figure 2, the green area indicates the reuse of a single filter stored in the weight BRAM. The red area (bottom right) shows the final reuse of weights from Weight BRAM_0, whereas the blue area shows the final reuse from Weight BRAM_$N_{W}$-1.

During convolution, each weight BRAM alternates between data reuse and new data loading while processing all the OFM channels. For example, once the computation in the red area is completed, Weight BRAM_0 loads the next weight segment from the memory, while the computation for the blue area proceeds. Similarly, after the computation in the blue area is finished, Weight BRAM_$N_{W}$-1 fetches the next weight segment, and the freshly loaded weights in Weight BRAM_0 are immediately used for the next OFM computation.

**Figure 3:**
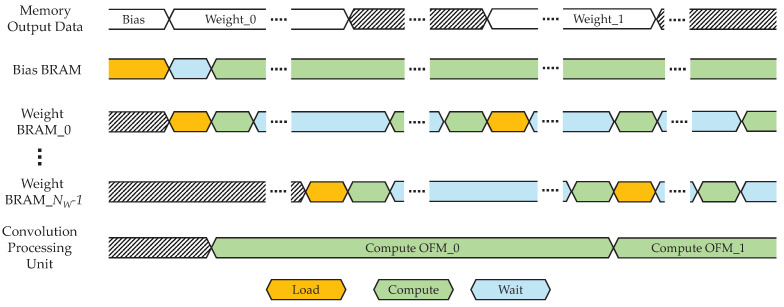
Timing diagram of BRAM operations for bias and weight data.

During convolution, each weight BRAM alternates between data reuse and new data loading while processing all the OFM channels. This alternating operation between data loading and computation is illustrated in the timing diagram in Figure 3. Once the bias BRAM is filled, the RDMA Controller sequentially loads weight segments, starting with weight BRAM_0. The loaded weight BRAM segments are alternately reused until the final OFM data has been computed. Accordingly, as each BRAM completes its reuse phase, it is sequentially reloaded with the next weight segment required for the subsequent OFM computation. This process is repeated until all OFM data for the entire layer has been generated.

### 3.3. IFM Reuse

We adopt an IFM reuse method that reduces the bandwidth by avoiding redundant memory access between the memory and PL for 3×3 convolution operations. This approach enables simultaneous data reuse and memory readings. If only the filter reuse structure (Section 3.2) is applied, the PU can quickly obtain valid weight data from the BRAM; however, IFM preparation is delayed owing to repeated memory accesses. Because a PU can operate only when both the weight and IFM data are available, a low-resource IFM reuse technique is essential to minimize redundant access and shorten the IFM preparation time.

**Figure 4:**
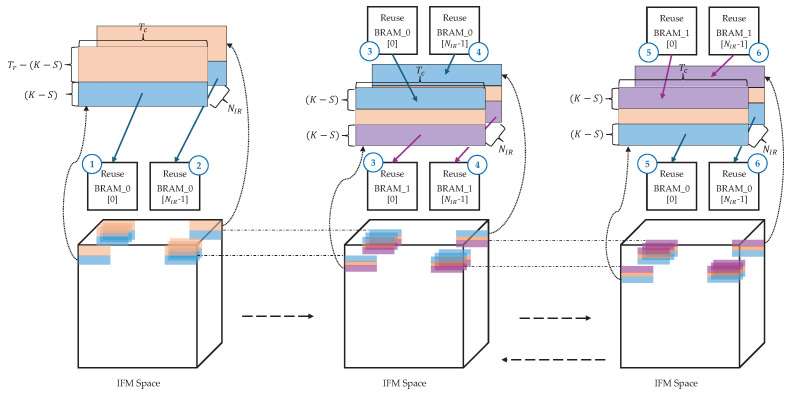
Illustration of the IFM data reuse method.

We address this by employing a line buffer-based IFM reuse strategy that stores $\left(K-S\right)\times T_{c}$-sized tiles—processed in the PU’s FFs—into BRAM for later reuse. As illustrated in Figure 4, the red area indicates IFM data that is not reused, the blue area corresponds to data read from or written to Reuse BRAM_0, and the purple area corresponds to data read from or written to Reuse BRAM_1.

(2) $$N_{IR}=\frac{I_{s}\times I_{c}}{T_{c}-\left(K-S\right)}.$$

The IFM reuse process operates as follows (Table 4):

Steps (③, ④), (⑤, and ⑥) are repeated until the processing of the final OFM row is complete, enabling efficient IFM data reuse. Afterward, the entire process repeats from step ① to process the next set of OFM data. Because this method reuses only part of a tile and stores it in BRAM rather than in local FFs, it reduces FF usage and places less demand on the overall hardware resources compared with fully local-stationary approaches.

### 3.4. Convolution Utilization

In this section, we determine the tiling parameters $T_{c}$ and $T_{r}$ to optimize resource utilization for the 3 × 3 convolution of the accelerator. The value of $T_{c}$ is first selected based on the convolution resource usage and the capacity of the IFM reuse BRAM, which is affected by $T_{c}$, as shown in (3). Once suitable $T_{c}$ candidates are identified, the final $T_{r}$ value is chosen by evaluating the corresponding DSP utilization.

(3) $$BRAM_{\mathrm{Reuse}}\left[\mathrm{Bytes}\right]=\left(K-S\right)\times N_{IR}\times T_{c}\times Q_{b}\times 2.$$

In this equation, the required BRAM size is primarily determined by the tiling column count ($T_{c}$), which is the parameter being optimized. The constant 2 represents the use of two BRAMs to enable double buffering.

In YOLOv2, the IFM size ($I_{s}$) varies across layers; consequently, a control scheme for the tiling column ($T_{c}$) that supports all possible tile configurations would incur substantial LUT overhead. To preserve a regular utilization structure while minimizing resource waste, the value of $T_{c}$ is determined based on $I_{s}$ with an additional padding of 2.

Table 5 summarizes the convolution resource utilization, the number of required IFM reuse BRAM addresses ($N_{IR}$), and the IFM reuse BRAM size for each $T_{c}$ value based on (2) and (3). For $T_{c}$ values of 418, 210, and 106, the convolution resource utilization drops to as low as 3.125% in layers where $I_{s}\geq 52$, indicating inefficient usage. By contrast, $T_{c}$ values of 54, 28, and 15 achieve utilization above 25%, demonstrating more effective resource usage. Conversely, smaller $T_{c}$ values generally increase both $N_{IR}$ and the required reuse BRAM size. The 153.6 KB required for the smallest candidate ($T_{c}=15$) remains well within the capacity of XC7Z020. Consequently, $T_{c}$ candidates should be chosen primarily based on convolution resource utilization. Considering that most primary computation layers in YOLOv2 have $I_{s}$ values of 26 or 13, $T_{c}=28$ and $T_{c}=15$ are selected as final candidates as both yield over 50% utilization for these layers.

In the proposed architecture, the preparation time of the 3 × 3 convolution PU ranges from one cycle (minimum) to ten cycles (maximum) depending on whether IFM reuse is applied. Therefore, a design that employs sufficient DSP resources to process all the tiles concurrently is preferred over one that sequentially processes tiles across multiple clock cycles. Note that, in the XC7Z020 device, the 16-bit quantized MAC operations can be efficiently mapped to a single DSP slice.

Accordingly, the number of multiply–accumulate operations ($MACs_{\mathrm{tile}}$) was calculated to determine the optimal DSP resource usage, as shown in Table 6. The MAC count for a single tile ($MACs_{\mathrm{tile}}$) is directly proportional to the number of tile rows ($T_{r}$) and columns ($T_{c}$), as indicated in (4).

(4) $$MACs_{\mathrm{tile}}=K^{2}\times \left(T_{c}-\left(K-S\right)\right)\times \left(T_{r}-\left(K-S\right)\right).$$

In addition, as shown in (1), the number of tiling $FFs_{\mathrm{tile}}$ also increases proportionally with $T_{r}$ and $T_{c}$. Therefore, both $MACs_{\mathrm{tile}}$ and $FF_{\mathrm{tile}}$ resource consumption must be considered when selecting the tiling parameters. Based on this, we analyzed $T_{r}$ values ranging from a minimum of 3 (the condition for a 3×3 convolution) up to 6 and observed that the $MACs_{\mathrm{tile}}$ counts remained relatively high for values up to $T_{r}=5$.

Notably, when $T_{c}=28$, the minimum $MACs_{\mathrm{tile}}$ count is 234, which already exceeds the 220 DSPs available on the XC7Z020 while also requiring a substantial number of FF resources. Although synthesis tool optimization could theoretically reduce DSP usage, in practice, such approaches often result in resource shortages or timing violations during the place-and-route stage [35]. Furthermore, because DSP resources are also required for operations such as Leaky ReLU activation and various controllers, we ultimately selected a tiling configuration of $T_{r}=3$ and $T_{c}=15$, which remains within the available 220 DSP budget of the XC7Z020.

**Figure 5:**
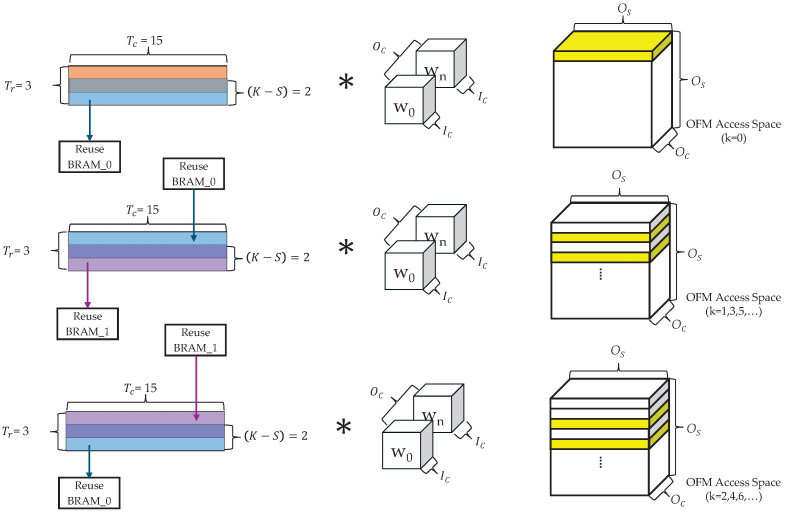
Tiling structure for a 3 × 3 convolution with $T_{r}=3$ and $T_{c}=15$.

The tiling structure for a 3×3 convolution with $T_{r}=3$ and $T_{c}=15$ is illustrated in Figure 5. In the initial computation phase for rows 0–2, the data for rows 1 and 2 are fetched from the external memory, whereas no memory access occurs for row 0 because it corresponds to a padding area. The fetched data from rows 1 and 2 are stored in the reuse BRAM. For subsequent computations (k>0), two reuse BRAMs are alternately used to process rows *k*, k+1, and k+2. During this process, the inputs for rows *k* and k+1 are read from one BRAM, whereas the results for rows k+1 and k+2 are written to the other BRAM, enabling continuous OFM computation.

The yellow area in Figure 5 indicates the region in which the corresponding tile contributes to OFM computation and does not represent the actual output order. The complete OFM is gradually generated by filling a space of size $O_{s}\times O_{s}$. This process is repeated along the $O_{c}$ dimension until the final output is produced.

### 3.5. Max Pooling Utilization

In this section, we determine the tiling parameters $T_{c_{mp}}$ and $T_{r_{mp}}$ to optimize resource utilization and data transfer efficiency for the max pooling operation of the accelerator.

Because max pooling does not involve data reuse, selecting tile sizes that achieve 100% hardware utilization is desirable from a resource perspective. However, if the number of pixels transferred per clock cycle is not an exact divisor of the tile width $I_{s}$, redundant non-valid pixels are sent alongside the valid data. This complicates pre-processing and increases memory access requirements, resulting in additional memory–PL latency and energy consumption. These inefficiencies can be avoided by selecting $T_{c_{mp}}$ and $T_{r_{mp}}$ to ensure that all transferred pixels are valid.

In this study, the AXI*x* burst size is configured to 8 bytes, enabling the transfer of four 16-bit pixels per clock cycle via RDMA. For max pooling layers where $I_{s}\geq 52$, the four-pixel transfer divides evenly into $I_{s}$, ensuring no redundant pixels in the final column. By contrast, for layers with $I_{s}=26$, two redundant pixels are transferred to the last column. This problem is addressed by selecting $T_{c_{mp}}=52$, which is the smallest multiple of 4 among the possible $I_{s}$ values. For the $I_{s}=26$ case, exceptions are handled by deactivating part of the tile during processing. Because YOLOv2 employs 2×2 max pooling, and the same pattern is repeated every two rows at $I_{s}=26$, $T_{r_{mp}}$ is set to 2.

**Figure 6:**
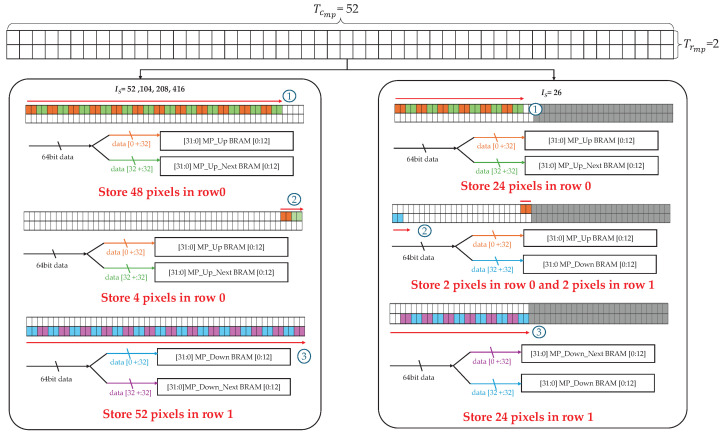
BRAM storage scheme for max pooling with $T_{r_{mp}}=2$ and $T_{c_{mp}}=52$.

Figure 6 illustrates the process of storing four 16-bit IFM pixels per clock cycle from memory into the BRAM for rows 0 and 1 using a little-endian format with $T_{r_{mp}}=2$ and $T_{c_{mp}}=52$. This storage strategy enables partial processing for cases where $I_{s}\geq 52$ and supports simple exception handling for $I_{s}=26$ through the following steps:

① For $I_{s}\geq 52$, regardless of the tile size, the first two pixels of the four transferred per cycle are stored in MP_Up BRAM, and the remaining two are stored in MP_Up_Next BRAM. This process continues until 48 pixels in row 0 are filled. For $I_{s}=26$, the same method is applied until 24 pixels of row 0 are stored.
② For $I_{s}\geq 52$, the remainder of row 0 is filled in the same manner as in Step ①. For $I_{s}=26$, however, the last two of the four pixels belong to the next row and are therefore stored in the MP_Down BRAM instead of the MP_Up_Next BRAM.
③ For $I_{s}\geq 52$, the first two pixels of the following row are stored in the MP_Down BRAM, and the subsequent two pixels are stored in MP_Down_Next BRAM. By contrast, for $I_{s}=26$, the storage order is reversed until the last column of row 1 is filled.

This sequence is repeated for rows 2 and 3 after the initial max pooling operation. Even-numbered rows (0, 2, 4, …) follow the procedures described in Steps ① and ②, whereas odd-numbered rows (1, 3, 5, …) follow Step ③.

**Figure 7:**
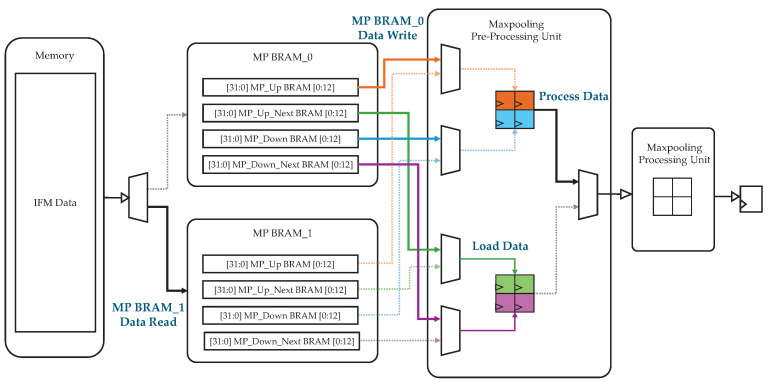
Max pooling architecture using $T_{r_{mp}}=2$ and $T_{c_{mp}}=52$.

Figure 7 presents the max pooling processing architecture, which employs two MP BRAM blocks (MP BRAM_0 and MP BRAM_1) configured using the BRAM storage scheme above. During operation, the two MP BRAMs are accessed alternately, enabling continuous read–write operations. Each BRAM comprises two pairs: {MP_Up BRAM, MP_Down BRAM} and {MP_Up_Next BRAM, MP_Down_Next BRAM}. The max pooling PU alternates between these two pairs in every clock cycle, processing one set of pixels while loading the next set in parallel. This parallelism allows four new pixels to be read from the memory in the same cycle in which max pooling is performed on another set of four pixels.

### 3.6. Acc _Lock Controller

This study adopts a stall-based mechanism that monitors the occupancy status of the OFM BRAMs in real time and pauses the pipeline operation when necessary.

**Figure 8:**
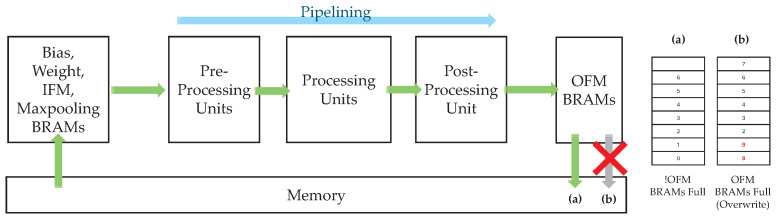
Accelerator architecture with potential structural hazards.

Figure 8 illustrates an accelerator architecture in which structural hazards may occur. The pre-processing units continuously receive data from the bias, weight, IFM, and max pooling BRAMs (hereafter referred to as BWIM BRAMs). Because the pre-processing units, processing units, and post-processing unit form a pipeline, the OFM BRAMs also receive a continuous stream of data. If both RDMA and WDMA maintain a stable flow, as shown in Figure 8a, the system produces a normal output. However, if the WDMA is temporarily stalled, as shown in Figure 8b, the subsequent pipeline outputs may overwrite the already filled OFM BRAMs, resulting in a structural hazard.

To address this issue, the proposed architecture incorporates an Acc_Lock controller that implements a stall-based control. The Acc_Lock controller directly monitors the occupancy level of the OFM BRAMs. When the storage ratio exceeds a predefined threshold, it generates an *Acc_Lock* signal.

**Figure 9:**
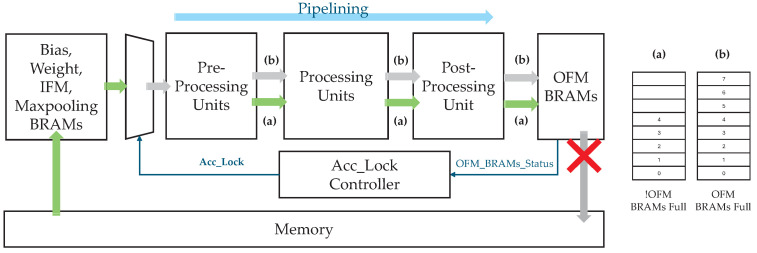
Accelerator architecture based on a stall mechanism.

As illustrated in Figure 9, the *Acc_Lock* signal functions as a switch between the BWIM BRAMs and the pre-processing units at the front end of the pipeline. When *Acc_Lock* is asserted, the data transfer from the BWIM BRAMs to the pre-processing units is halted, and only the data already present in the pipeline are processed (Figure 9a). This ensures that, as shown in Figure 9b, the OFM BRAMs remain full without being overwritten, thereby allowing the pipeline to stably process its internal data without new input.

Even during the stall period, the BWIM BRAMs can continue to fetch data internally via RDMA until they are fully loaded. Once the WDMA resumes and the OFM BRAM occupancy drops below the threshold, *Acc_Lock* is deactivated, and the pipeline returns to normal operation. This stall-based mechanism prevents output-path bottlenecks from affecting the entire system and ensures a lossless pipeline operation.

### 3.7. Proposed Accelerator

This section presents the overall architecture of the proposed YOLOv2 accelerator, describing its main hardware components, dataflow organization, and communication interfaces. The objective is to clarify how computation and control are distributed across the system and how data moves between the PS, external memory, and PL.

**Figure 10:**
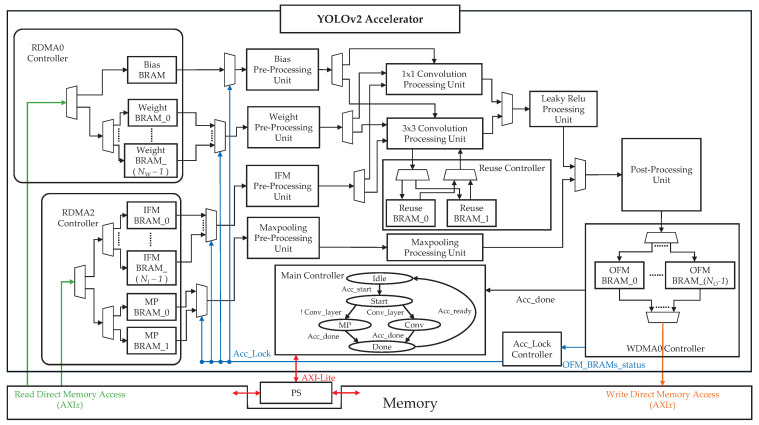
Overall architecture of the proposed accelerator.

The complete architecture is illustrated in Figure 10. The architecture is organized into two main component groups—controllers and PUs—which communicate with the PS and external memory through the AXI-Lite and AXI*x* interfaces.

The accelerator contains six controllers and nine PUs. The controllers manage the DMA transfers (RDMA0, RDMA2, and WDMA0), coordinate the overall accelerator operation (main controller and Acc_Lock controller), and control the reuse BRAM (reuse controller). The PUs perform the pre-processing, computation, and post-processing required for the convolution and max pooling layers. Each processing unit adopts a pipelined structure to enable continuous data output.

Only K=2 operations are required for max pooling; hence, a single max pooling PU is implemented. Convolution requires K=1 and K=3 operations; therefore, two convolution PUs are included. The 3×3 convolution PU applies the IFM reuse method (Section 3.3) with the tiling parameters $T_{r}=3$ and $T_{c}=15$ (Section 3.4). The 1×1 convolution PU operates with $T_{r}=1$ and $T_{c}=13$, matching the 13 outputs per cycle of the 3×3 unit in the S=1 case of YOLOv2 to simplify post-processing. IFM reuse is not applied to 1×1 convolution because no redundant IFM access occurs within a single OFM computation.

The accelerator employs a primary controller that manages data movement, computation sequencing, and pipeline flow control to coordinate these heterogeneous units. This is achieved through an FSM that defines the distinct operational stages and transitions between them. Clearly specifying the FSM states and transitions is essential for understanding how the configuration parameters from the PS are translated into synchronized hardware actions across all controllers and PUs. Furthermore, several hardware parameters can be configured to adapt the accelerator to different layer dimensions and memory bandwidth constraints.

The remainder of this section details the FSM states of the main controller, the FSM state transition behavior, and the configurable hardware parameters that influence resource allocation and execution flow.

#### 3.7.1. Main Controller FSM States

The FSM of the main controller governs the operation of all controllers and PUs, ensuring correct sequencing and dataflow coordination. Table 7 summarizes each state and its function.

#### 3.7.2. FSM State Transitions

FSM transitions follow a deterministic sequence driven by control signals and task completion events.

1. **Idle → Start:** Triggered when Acc_start is received from the PS via AXI-Lite.
2. **Start → MP/Conv:** Determined by Conv_layer; 0 for MP, 1 for Conv.
3. **MP/Conv → Done:** Triggered when WDMA0 completes data transfers and issues Acc_done.
4. **Done → Idle:** Triggered when all units are ready and Acc_ready is asserted.

#### 3.7.3. Configurable Hardware Parameters

Several hardware parameters can be adjusted at the design stage to balance the resource usage, memory bandwidth, and performance.

- $N_{W}$: Number of weight BRAMs. Increasing $N_{W}$ reduces the initial computational latency by enabling faster weight loading.
- $N_{I}$: Number of IFM BRAMs. Increasing $N_{I}$ reduces latency by overlapping IFM loading with weight/bias loading and allows IFM prefetching during reuse-only computation phases for 3×3 convolution.
- $N_{O}$: Number of OFM BRAMs. Increasing $N_{O}$ reduces the probability of Acc_Lock activation, thereby lowering stall-induced latency.

## 4. FPGA Evaluation

This section presents the implementation process and results of the proposed accelerator architecture at the register-transfer level (RTL). The design was deployed on a Zybo-Z7-20 board [36], which integrates an XC7Z020 SoC FPGA with a dual-core ARM Cortex-A9 processor. The accelerator architecture shown in Figure 10 was fully described in Verilog RTL and integrated into a Linux-based execution environment to run the YOLOv2 model.

Synthesis and implementation were performed using Xilinx Vivado 2023.2, and PetaLinux 2023.2 was employed to configure the Linux operating system environment. The main hardware parameters used in the RTL design were set as $N_{W}=16$, $N_{I}=12$, and $N_{O}=3$.

For validation, we developed a C-based Accelerator_Test (Acc_test) application using the COCO [3] and UA-DETRAC datasets [37] and applied the post-training quantization (PTQ) method. The INT16-quantized data generated via PTQ were used to evaluate the object detection accuracy of the accelerator. Additionally, these data were used to measure the end-to-end processing time, from image input to the final bounding box output.

### 4.1. RTL Synthesis and Implementation

**Figure 11:**
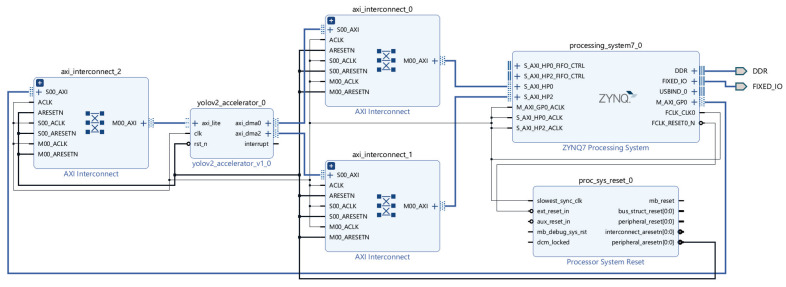
Block design of the YOLOv2 accelerator connected to the Zynq PS.

The Zybo Z7-20 board, equipped with the XC7Z020 SoC, supports DMA via the AXI3 protocol. As illustrated in Figure 11, the control signals are transferred from the PS to the accelerator through the GP0 port via AXI-Lite communication. The accelerator (yolov2_accelerator_0) performs the DMA operations via axi_dma0, connected to the HP0 port, and axi_dma2, connected to the HP2 port.

The bias and weight data are read using RDMA through the HP0 port. As these filters are reused, no additional memory access is required until the weight BRAM reuse cycle is complete. In addition, the OFM data for the WDMA is accessed through the HP0 port to ensure efficient memory utilization. By contrast, RDMA for IFM data involves frequent memory access; therefore, assigning a dedicated port is more efficient. In this implementation, the HP2 port was used instead of the HP1 port because the HP0 and HP1 ports share the same interconnect path [38].

**Figure 12:**
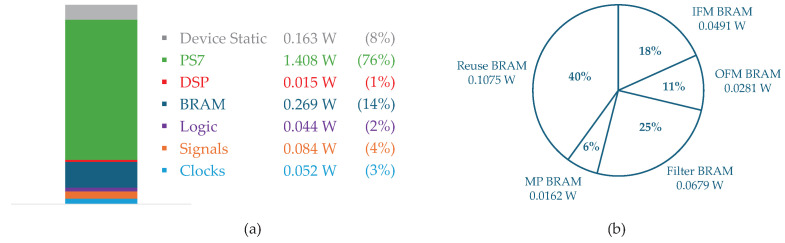
Total power and BRAM power consumption.

Table 8 presents the implementation results at 100 MHz, which is the maximum achievable PL clock frequency for synthesis and implementation on the XC7Z020. The proposed accelerator achieved 49.15% LUT, 16.55% FF, 70.00% BRAM, and 57.27% DSP utilization, confirming that the design fits within the resource constraints of the XC7Z020 SoC. After implementation, the total power consumption of the SoC was 2.035 W. As shown in Figure 12a, the Zynq-7000 PS7 was the largest contributor, consuming 1.408 W. The BRAMs were the second-highest power consumer at 0.269 W, and a detailed breakdown is provided in Figure 12b. Among the BRAMs, the reuse BRAMs accounted for the largest portion of this consumption at 0.1075 W (40%), while the MP BRAMs represented the smallest portion at 0.0162 W (6%).

### 4.2. System-Level Evaluation

In this study, we developed an Acc_test program in C to process the convolution and max pooling layers of YOLOv2 at the system level using the proposed accelerator. A Linux environment and a compiler are required to build this code into an object file on the Zynq-7000 SoC. PetaLinux, a Yocto-based embedded Linux distribution, allows the root file system (rootfs) to be configured using only the necessary build tools [39]. Therefore, the GCC compiler (petalinux-build-essential), GCC runtime, and make utility were added to the PetaLinux rootfs configuration before building the system to generate the Acc_test.o object file.

When executed, the generated Acc_test.o file performs object detection on 416×416×3 images using the YOLOv2 configuration file (yolov2.cfg) [40]. During this process, convolution and max pooling operations are executed in the PL region, whereas the concatenation and detection layers are processed in the PS region on the dual-core ARM Cortex-A9.

**Figure 13:**
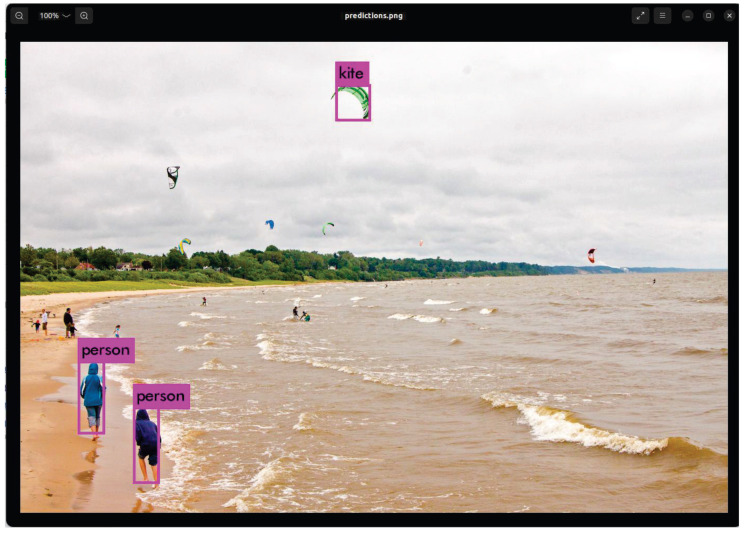
Final detection image obtained using the accelerator (INT16).

After completing all layer operations and bounding box generation, Acc_test.o outputs the final detection image (Figure 13) for the COCO dataset, along with the processing time and detection accuracy. The processing time for layers up to the detection layer is approximately 12.02 s, whereas the total time including bounding box drawing is approximately 12.64 s. Furthermore, only a negligible accuracy difference of approximately 0.2% exists between running the model with FP32 on a host PC [40] (kite: 64.7%, person: 58.1%, and person: 57.7%) and running it on the accelerator with INT16 quantization (kite: 64.9%, person: 58.3%, and person: 57.9%).

**Figure 14:**
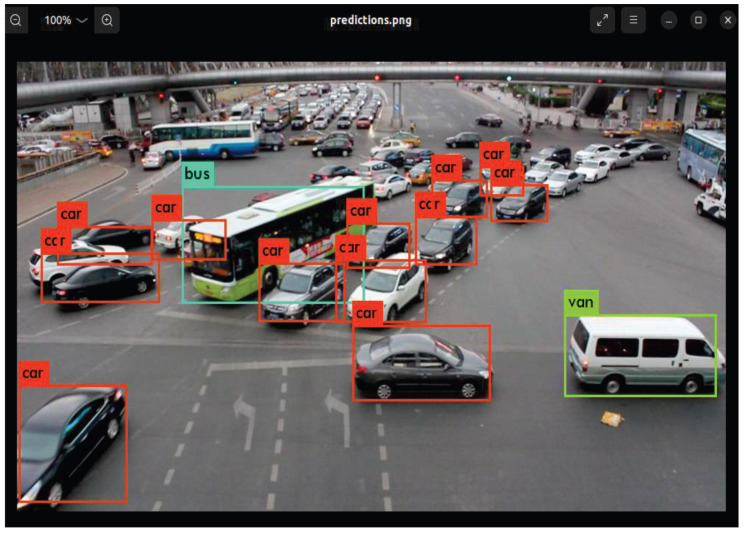
Final detection image obtained using the accelerator (INT16, UA-DETRAC dataset).

Figure 14 illustrates the detection results after training on the UA-DETRAC dataset. The accelerator’s total inference time to generate bounding boxes was approximately 12.42 s, with the feature extraction layers (prior to the detection layer) accounting for 11.80 s. In terms of accuracy, the INT16 accelerator consistently outperformed the FP32 host PC by a margin of 0.2% across all classes, a trend also observed with the COCO dataset. The detailed mAP results are as follows: host PC (bus: 93.4%, van: 89.5%, car-max: 90.8%, and car-min: 54.1%) and accelerator (bus: 93.6%, van: 89.7%, car-max: 91.0%, and car-min: 54.3%). The difference in computation time is a direct result of the reduced number of filters in the final 1×1 convolution layer, which decreased from 425 for the COCO dataset to 45 for the UA-DETRAC dataset.

### 4.3. Comparison with Other FPGA Implementations

Table 9 summarizes the resource utilization, throughput, and power consumption of the proposed accelerator compared with those of a conventional YOLOv4-Tiny accelerator [41], a YOLOv3-Tiny accelerator [42], and a YOLOv3 accelerator [43], all implemented on the same XC7Z020 SoC. In addition, we compare our design with a YOLOv2 accelerator [44] implemented on a different SoC, the XCZU9EG. The comparative analysis is as follows.

In [41], owing to the diverse layer structures of YOLOv4, multiple operational modules must be implemented in the PL, whereas the remaining operations are executed in the PS. This results in higher resource usage and lower GOPS performance. In particular, FF and BRAM utilization are approximately 12% and 21% higher, respectively, than those in our study. The work in [42] achieved a higher GOPS than our architecture by supporting parallel processing as well as upsampling and concatenation operations in the hardware. However, this results in the FF and DSP utilization being approximately 26% and 15% greater, respectively, than that in our design. In [43], GOPS performance was improved by adopting *im2col* and GEMM-based matrix multiplication instead of a sliding-window approach; however, this led to high resource usage. In particular, the LUT, FF, and BRAM utilization were approximately 22%, 23%, and 21% higher, respectively, than those in our study. Compared with [44], our architecture achieves lower LUT, FF, and BRAM utilization since their design supports parallel processing.

Overall, although the proposed accelerator may have some limitations in terms of functionality and computational performance compared with previous designs, it offers the advantage of implementing a YOLO accelerator architecture with significantly lower resource requirements.

### 4.4. Remarks

Overall, the proposed accelerator is capable of implementing a YOLO-based architecture with substantially reduced resource requirements. In particular, it decreases DSP and FF utilization by up to 15% and 26%, respectively, compared with other designs on the same FPGA board. When compared with [41], which demonstrates the lowest LUT and BRAM consumption among prior works, our design achieves a comparable level of LUT usage while exhibiting only a marginal 4% increase in BRAM utilization. This confirms that the reduction in DSP and FF resources is not achieved through a simple trade-off that would otherwise increase LUT or BRAM usage. Consequently, consistent with the design objective of enabling deployment in resource-constrained environments, the proposed accelerator demonstrates a clear advantage in overall resource efficiency.

Despite its efficiency in resource utilization, the proposed architecture exhibits limitations with respect to latency. The primary cause of this latency is the absence of parallel processing. Unlike the designs presented in [42,43], our architecture does not adopt multi-filter parallelism within the sliding-window structure, which results in lower throughput compared to previous accelerators. In addition, the computational workload of our model is approximately 3.9×, 5.3×, and 1.5× higher than those reported in [41,42,43], respectively, thereby proportionally increasing the computation time.

## 5. Conclusions

This study presents an XC7Z020-based YOLOv2 accelerator architecture optimized for the XC7Z020 (53200 LUT, 106400 FF, 220 DSP, 140 BRAM). INT16 quantization was applied to minimize energy consumption and latency by reducing memory–PL bandwidth, and filter reuse and IFM reuse techniques were adopted to reuse large-volume data during convolution efficiently. Furthermore, the tiling parameters for the convolution and max pooling operations were carefully selected based on an analysis of the resource structure and utilization of XC7Z020, thereby reducing inefficient resource usage. A stall-based Acc_Lock controller was incorporated to prevent structural hazards. The proposed architecture was synthesized and implemented on a Zybo Z7-20 board, demonstrating lower resource utilization compared with previous accelerators. System-level evaluation in a real Linux environment further confirmed that the detection accuracy shows only a negligible difference from the FP32 baseline.

In future work, the performance can be further improved by exploring multi-filter computation using an input-stationary approach during convolution operations to maximize parallelism. In addition, the architecture can be extended to support more complex models by incorporating additional acceleration structures, such as an upsample module. Furthermore, this study can be extended to the latest YOLO models, demonstrating the scalability of the proposed design toward more advanced object detection frameworks.

## Figures and Tables

**Table 1:** Definitions of the variables.

Variables	Definition
$I_{s}$	Size of IFM (13/26/52/104/208/416)
$I_{c}$	Size of IFM channel
$O_{s}$	Size of OFM
$O_{c}$	Size of OFM channel
$Q_{b}$	Size of quantization in bytes [Bytes]
$B_{c}$	Bias channel size
*K*	Kernel size
*S*	Stride size
$N_{F}$	Number of filters
$N_{IR}$	Number of IFM reuse BRAM addresses
$N_{W}$	Number of weight BRAMs
$N_{I}$	Number of IFM BRAMs
$N_{O}$	Number of OFM BRAMs
$T_{r}$	Size of tiles in the row direction
$T_{c}$	Size of tiles in the column direction
$T_{r_{mp}}$	Size of tiles in the row direction for max pooling
$T_{c_{mp}}$	Size of tiles in the column direction for max pooling
$FF_{\mathrm{tile}}$	Number of flip-flops required for the tile storage
$BRAM_{\mathrm{Reuse}}$	Capacity of the IFM reuse BRAM [Bytes]
$MACs_{\mathrm{tile}}$	The MAC count for a single tile

**Table 2:** Definitions of FPGA hardware resource abbreviations.

Abbreviation	Definition
LUT	Look-Up Table
FF	Flip-Flop
BRAM	36 Kb Block Random Access Memory
DSP	Digital Signal Processing

**Table 3:** Maximum possible sizes of IFM, bias, and weight.

	Maximum IFM (Bytes)	Maximum Bias (Bytes)	Maximum Weight (Bytes)
Required bytes	$I_{s}^{2}\times I_{c}\times Q_{b}$	$B_{c}^{2}\times Q_{b}$	$N_{F}\times K^{2}\times I_{c}\times Q_{b}$
32 bit	$416^{2}\times 32\times 4$ (22.15 MB)	1280×4 (5.12 KB)	$1024\times 3^{2}\times 1280\times 4$ (47.19 MB)
16 bit	$416^{2}\times 32\times 2$ (11.08 MB)	1280×2 (2.56 KB)	$1024\times 3^{2}\times 1280\times 2$ (23.59 MB = 1024 × 23.04 KB)

**Table 4:** State transition of the IFM reuse BRAM.

Steps	Processing Step (IFM Row)	PU Data Source	Reuse BRAM_0 ^b^ State	Reuse BRAM_1 ^b^ State	Number of IFM Data Stored
①	**P ^a^ ← 0;** 0 to $T_{r}-1$	Memory	Read	Idle	(K−S) × $T_{c}$
②	0 to $T_{r}-1$	Memory	Read	Idle	(K−S) × $T_{c}\times N_{IR}$
③	**P ← P + 1;** **P** × ($T_{r}-\left(K-S\right)$) to **P** × ($T_{r}-\left(K-S\right))$ + $T_{r}-1$	Memory + BRAM_0	Write	Read	(K−S) × $T_{c}$
④	**P** × ($T_{r}-\left(K-S\right)$) to **P** × ($T_{r}-\left(K-S\right)$) + $T_{r}-1$	Memory + BRAM_0	Write	Read	(K−S) × $T_{c}\times N_{IR}$
⑤	**P ← P + 1;** **P** × ($T_{r}-\left(K-S\right)$) to **P** × ($T_{r}-\left(K-S\right)$) + $T_{r}-1$	Memory + BRAM_1	Read	Write	(K−S) × $T_{c}$
⑥	**P** × ($T_{r}-\left(K-S\right)$) to **P** × ($T_{r}-\left(K-S\right)$) + $T_{r}-1$	Memory + BRAM_1	Read	Write	(K−S) × $T_{c}\times N_{IR}$

^a^ The variable **P** determines the range of rows currently being processed. ^b^ Read and write represent ‘read data from tiles’ and ‘write data to tiles’.

**Table 5:** Convolution resource utilization according to Tc and the number and size of the IFM reuse BRAM.

	$I_{s}\times I_{s}\times I_{c}$	$T_{c}=418$	$T_{c}=210$	$T_{c}=106$	$T_{c}=54$	$T_{c}=28$	$T_{c}=15$
Convolution Resource Utilization [%]	416×416×3	100%					
	208×208×32	50%	100%				
	104×104×64	25%	50%	100%			
	52×52×128	12.5%	25%	50%	100%		
	26×26×256	6.25%	12.5%	25%	50%	100%	
	13×13×1280	3.125%	6.25%	12.5%	25%	50%	100%
Maximum $N_{IR}$ $\left(I_{s}=13,I_{c}=1280\right)$		40	80	160	320	640	1280
${\mathrm{BRAM}}_{\mathrm{Reuse}}$		133.76 KB	133.4 KB	135.68 KB	138.24 KB	143.36 KB	153.6 KB

**Table 6:** Required $MACs_{\mathrm{tile}}$ and $FF_{\mathrm{tile}}$ counts according to $T_{r}$ for $T_{c}$ = 15 and $T_{c}$ = 28.

	$T_{r}$							
	**3**		**4**		**5**		**6**	
	${\mathrm{MACs}}_{\mathrm{tile}}$	${\mathrm{FF}}_{\mathrm{tile}}$	${\mathrm{MACs}}_{\mathrm{tile}}$	${\mathrm{FF}}_{\mathrm{tile}}$	${\mathrm{MACs}}_{\mathrm{tile}}$	${\mathrm{FF}}_{\mathrm{tile}}$	${\mathrm{MACs}}_{\mathrm{tile}}$	${\mathrm{FF}}_{\mathrm{tile}}$
$T_{c}=15$	117	720	234	960	351	1200	468	1440
$T_{c}=28$	234	1344	468	1792	702	2240	936	2688

**Table 7:** FSM states of the main controller and their operations.

State	Name	Description
1	Idle	Initial state of the accelerator. The main controller receives the layer configuration and Acc_start signal from the PS via AXI-Lite.
2	Start	Preparation stage. Based on the received layer information, the main controller generates control data and issues signals to other controllers and PUs.
3-1	MP	Max pooling stage. The RDMA2 controller loads IFM data into MP BRAMs, which are processed through the max pooling pre-processing unit, max pooling processing unit, and post-processing unit. OFM data are grouped into 64-bit packets, stored in OFM BRAMs, and then transferred to external memory via WDMA0. The Acc_Lock controller monitors OFM BRAM occupancy to manage pipeline flow.
3-2	Conv	Convolution stage. The RDMA0 controller loads bias and weight data into their respective BRAMs, while the RDMA2 controller loads IFM data. Pre-processed data are sent to either the 1×1 or 3×3 convolution PU, followed by the Leaky ReLU PU and post-processing unit. Processed OFM data are grouped, stored in OFM BRAMs, and transferred to memory via WDMA0. Pipeline flow is regulated by the Acc_Lock controller.
4	Done	Completion stage. The main controller sends Acc_done to the PS via AXI-Lite and resets all units in preparation for the next layer.

**Table 8:** Synthesis and implementation results.

Resource	Used	Available	Utilization [%]
LUT	26,147	53,200	49.15%
FF	17,605	106,400	16.55%
BRAM	98	140	70.00%
DSP	126	220	57.27%

**Table 9:** Comparison with the previous YOLO accelerator.

	[41]	[42]	[43]	[44]	This Work
Target FPGA	ZedBoard	ZedBoard	Zynq-7000 SoCs	ZCU 102	Zybo-Z7-20
Model	YOLOv4-Tiny	YOLOv3-Tiny	YOLOv3	YOLOv2	YOLOv2
Dataset	COCO	COCO	UA-DETRAC	COCO	COCO
mAP (%)	40.2	30.9	71.1	48.1	48.1
Model GFLOPs	7.5	5.6	19.5	29.5	29.5
LUT	31 K (58%)	26 K (49%)	38 K (71%)	95 K (35%)	26 K (49%)
FF	31 K (29%)	46 K (43%)	43 K (40%)	90 K (17%)	**17 K (17%)**
BRAM (36 Kb)	132 (94%)	92.5 (66%)	132.5 (94%)	245.5 (27%)	98 (70%)
DSP	149 (67%)	160 (72%)	144 (65%)	609 (24%)	**126 (57%)**
Frequency [MHz]	100	100	230	300	100
Latency [ms]	18,025	532	310	288	12,639
GOPS ^1^	0.4	10.5	62.9	102.43	2.33
GOPS/DSP	0.003	0.07	0.437	0.168	0.018
Energy [mJ] ^2^	42,900	1787.52	471.2	3398.4	25,720.37
Power [W]	1.994	3.4	1.52	11.8	2.035

^1^ GOPS = (Model GFLOPs)/(Latency [ms]/1000). ^2^ Energy = Power [W] × Latency [ms].

## Data Availability

The data presented in this study are available on request from the corresponding author.
